# Association between the cytokine storm, immune cell dynamics, and viral replicative capacity in hyperacute HIV infection

**DOI:** 10.1186/s12916-020-01529-6

**Published:** 2020-03-25

**Authors:** Daniel M. Muema, Ngomu A. Akilimali, Okechukwu C. Ndumnego, Sipho S. Rasehlo, Raveshni Durgiah, Doty B.A. Ojwach, Nasreen Ismail, Mary Dong, Amber Moodley, Krista L. Dong, Zaza M. Ndhlovu, Jenniffer M. Mabuka, Bruce D. Walker, Jaclyn K. Mann, Thumbi Ndung’u

**Affiliations:** 1 Africa Health Research Institute, Durban, South Africa; 2 HIV Pathogenesis Programme, The Doris Duke Medical Research Institute, University of KwaZulu-Natal, Durban, South Africa; 3 KEMRI-Wellcome Trust Research Programme, Kilifi, Kenya; 4 Ragon Institute of MGH, MIT and Harvard University, Cambridge, MA, USA; 5 Max Planck Institute for Infection Biology, Berlin, Germany; 6 Division of Infection and Immunity, University College London, London, UK

**Keywords:** Acute HIV infection, Cytokine storm, Early ART, Replication capacity

## Abstract

**Introduction:**

Immunological damage in acute HIV infection (AHI) may predispose to detrimental clinical sequela. However, studies on the earliest HIV-induced immunological changes are limited, particularly in sub-Saharan Africa. We assessed the plasma cytokines kinetics, and their associations with virological and immunological parameters, in a well-characterized AHI cohort where participants were diagnosed before peak viremia.

**Methods:**

Blood cytokine levels were measured using Luminex and ELISA assays pre-infection, during the hyperacute infection phase (before or at peak viremia, 1–11 days after the first detection of viremia), after peak viremia (24–32 days), and during the early chronic phase (77–263 days). Gag-protease-driven replicative capacities of the transmitted/founder viruses were determined using a green fluorescent reporter T cell assay. Complete blood counts were determined before and immediately following AHI detection before ART initiation.

**Results:**

Untreated AHI was associated with a cytokine storm of 12 out of the 33 cytokines analyzed. Initiation of ART during Fiebig stages I–II abrogated the cytokine storm. In untreated AHI, virus replicative capacity correlated positively with IP-10 (rho = 0.84, *P* < 0.001) and IFN-alpha (rho = 0.59, *P* = 0.045) and inversely with nadir CD4^+^ T cell counts (rho = − 0.58, *P* = 0.048). Hyperacute HIV infection before the initiation of ART was associated with a transient increase in monocytes (*P* < 0.001), decreased lymphocytes (*P* = 0.011) and eosinophils (*P* = 0.003) at Fiebig stages I–II, and decreased eosinophils (*P* < 0.001) and basophils (*P* = 0.007) at Fiebig stages III–V. Levels of CXCL13 during the untreated hyperacute phase correlated inversely with blood eosinophils (rho = − 0.89, *P* < 0.001), basophils (rho = − 0.87, *P* = 0.001) and lymphocytes (rho = − 0.81, *P* = 0.005), suggesting their trafficking into tissues. In early treated individuals, time to viral load suppression correlated positively with plasma CXCL13 at the early chronic phase (rho = 0.83, *P* = 0.042).

**Conclusion:**

While commencement of ART during Fiebig stages I–II of AHI abrogated the HIV-induced cytokine storm, significant depletions of eosinophils, basophils, and lymphocytes, as well as transient expansions of monocytes, were still observed in these individuals in the hyperacute phase before the initiation of ART, suggesting that even ART initiated during the onset of viremia does not abrogate all HIV-induced immune changes.

## Background

HIV infection is associated with immune activation which manifests in the elevation of numerous plasma cytokines and chemokines [1–7]. Notably, the elevation of some plasma cytokines occurs very early after infection, with acute HIV infection (AHI) being characterized by the onset of a cytokine storm in the period leading up to peak viremia [1, 2, 4, 6]. The resolution of the cytokine storm following peak viremia is partial, with some cytokines remaining elevated above pre-infection physiologic levels which persist into the chronic phase in untreated infection. Similar observations of the cytokine storm have been reported in acute pathogenic SIV infections in rhesus macaques [8–11]. Even though the initiation of ART significantly reduces the plasma levels of inflammatory cytokines, some residual immune activation has been reported to persist even after the early initiation of ART [7, 12, 13].

AHI is also associated with dysregulations in lymphocyte compartments [6, 14–16]. Partial spontaneous recovery of some of the lymphocyte compartments occurs after peak viremia [14, 15]. Early initiation of ART in AHI allows the recovery of helper CD4^+^ T cells [15]. The effects of AHI on other white blood cells, especially in the granulocytes compartment, are poorly understood.

The HIV-induced inflammatory immune activation is linked to poor outcomes in chronic HIV infection due to its association with CD4^+^ T cell depletion, higher viral replication, and increased risk of non-immunological complications including cardiovascular complications and kidney dysfunction [10, 17–22]. Even though most HIV-associated complications only become clinically apparent during the chronic phase of infection, the nature of the early cytokine profile could set the tempo for later immune-driven pathologies. For example, in SIV infection models, high levels of inflammatory cytokines in the acute phase are associated with faster disease progression and higher viral loads during chronic phases [11]. However, early immunologic and virologic events in HIV infection, and their impact on subsequent disease progression, remain understudied due to the logistical difficulties in obtaining pre-infection and early acute infection samples from HIV-infected individuals. Most human immunological studies during AHI have been done in resource-rich countries in patients infected with HIV subtype B, yet HIV-1 subtypes differ in their virulence [1, 23–25]. Also, the previous studies were mostly conducted in cohorts that were dominated by male patients, yet the highest burden of HIV in sub-Saharan Africa is in women, and gender has been shown to affect inflammatory responses [26]. Furthermore, geographical localization determines the basal inflammatory state due to environmental exposure to other endemic pathogens [27]. As such, HIV-1 subtype C, which is the most common clade in Southern Africa where women are most affected, may induce a cytokine storm that drives unique pathologies [24, 25].

A better understanding of how HIV-1 subtype C infection in women in sub-Saharan Africa perturbs the immune environment during AHI, and how this may potentially lead to immune-driven pathologies and clinically significant sequela, is necessary. This will inform strategies to modulate immune responses and confer clinical benefits in vaccine, treatment, and cure efforts. Such knowledge may also guide efforts to mitigate HIV-associated co-morbidities, such as cardiovascular events and kidney dysfunction, in HIV-1 subtype C infections. Here, we longitudinally investigated the levels of plasma cytokines in acutely infected early-treated and untreated young women identified in a well-characterized hyperacute HIV-1 subtype C infection cohort, in whom pre-infection and longitudinal post-infection samples were available [15]. We also assessed the early immune cell dynamics in untreated acute HIV in the entire cohort by comparing pre-infection complete blood counts with respective measurements at the first post-infection sampling time point before the initiation of treatment. Further, we checked for correlations between the cytokines and virological and immunological parameters to determine their possible associations. We show that even though the cytokine storm that is induced in acute HIV-1 subtype C infection is abrogated by immediate treatment, some cellular changes still occur in all individuals before the earliest possible initiation of treatment. We also show novel associations between the plasma cytokines and virological and immunological parameters, providing insight into the pathogenesis of acute HIV-1 subtype C.

## Methods

### Study participants

HIV-uninfected women aged 18–23 years were recruited into the Females Rising through Education, Support, and Health (FRESH) cohort in Umlazi, Durban, South Africa [15]. Participants attended a twice-weekly socioeconomic empowerment program which was integrated with the study protocol. At each visit, a finger-prick blood draw was performed for HIV RNA testing. Blood samples were collected and archived every 3 months during the surveillance/empowerment period. From the participants who tested positive for HIV RNA, biological sampling continued regularly, during the acute and chronic phases.

In accordance with the South African national clinical guidelines at that time, treatment was deferred in the first 14 participants identified with AHI until CD4^+^ T cells declined to the recommended eligibility threshold which was changed from 350 to 500 cells/μL during that period. Subsequently, the study protocol was modified, enabling all participants who tested positive for HIV RNA to be initiated on immediate ART. Complete blood counts (CBCs), CD4^+^ T cell counts, and viral loads were determined at an accredited diagnostic laboratory in Durban, South Africa. CBCs were determined in the FRESH participants before infection and at the earliest post-infection visit, before the initiation of ART. All infected participants in the FRESH cohort were infected with HIV-1 subtype C.

For comparison, measurements from HIV-1 subtype C-infected chronic viremic participants in a separate cohort, the HIV Pathogenesis Programme (HPP) acute infection cohort in Durban, South Africa, were included in some of the analyses [28]. In this cohort, CBCs had been determined in some untreated participants at the chronic phase of infection before the initiation of ART as per the clinical guidelines at that time.

### Sample processing and storage of plasma

Blood samples were collected in acid citrate dextrose tubes (BD, Franklin Lakes, NJ, USA) and immediately transported to the laboratory. The plasma component was separated from the cellular component by centrifugation within 6 h, followed by immediate storage at − 80 °C. To avoid repeated freeze-thaw cycles that could negatively impact the cytokines, at the time of the assays, the plasma samples were thawed once and aliquoted into 96-well microplates that were sealed and stored at − 80 °C for future cytokines assays.

### Determination of cytokine concentrations in plasma

Plasma concentrations for 30 cytokines and markers of inflammation were measured using a 30-plex Luminex kit (Invitrogen, Carlsbad, CA, USA) at the following stages: pre-infection (12–150 days before the detection of viremia), during the hyperacute infection phase (4–11 days after the detection of viremia), after peak viremia (24–32 days after the detection of viremia), and during the early chronic phase (238–263 days [except one participant at 434 days] after the detection of viremia). The kit targeted the following analytes: FGF-Basic, IL-1beta, G-CSF, IL-10, IL-13, IL-6, IL-12, RANTES, eotaxin, IL-17, MIP-1alpha, GM-CSF, MIP-1beta, MCP-1, IL-15, EGF, IL-5, HGF, VEGF, IFN-gamma, IFN-alpha, IL-1RA, TNF-alpha, IL-2, IL-7, IP-10, soluble IL-2 receptor, MIG, IL-4, and IL-8. In addition, plasma IFN-alpha was measured pre-infection, at two time points in the hyperacute phase (1–4 days and 4–11 days after the detection of viremia) and at two time points after peak viremia (13–18 days and 24–32 days after the detection of viremia) using the human IFN-alpha multi-subtype ELISA kit (PBL Assay Science, Piscataway, NJ, USA). Since the measurements of IFN-alpha were done with both the Luminex kit and the ELISA kit, the ELISA measurements were chosen for analyses because they had additional time points during AHI (1–4 days and 13–18 weeks) that were not captured in the Luminex measurements. The plasma levels of BAFF had been measured in a previous study pre-infection, during the hyperacute infection phase (4–11 days after the detection of viremia), at two time points after peak viremia (13–18 days and 24–32 days after the detection of viremia), and during the early chronic phase (77–95 days after the detection of viremia) using Human BAFF Quantikine ELISA kit (R&D Systems, Minneapolis, MN, USA) while CXCL13 had been measured pre-infection, during the hyperacute infection phase (1–4 days after the detection of viremia), at two time points after peak viremia (13–18 days and 24–32 days after the detection of viremia), and during the early chronic phase (77–95 days after the detection of viremia) using human CXCL13 Quantikine ELISA kit (R&D Systems) [6]. Soluble CD14 was measured using human CD14 DuoSet ELISA pre-infection, during the hyperacute phase (4–11 days after the detection of viremia), after peak viremia (24–32 days after the detection of viremia), and during the early chronic phase (238–263 days [except one participant at 434 days] after the detection of viremia) (R&D Systems).

### Determination of HIV replication capacity

Gag-protease recombinant viruses were generated as previously described [29]. Briefly, viral RNA was extracted from acute infection plasma samples. Reverse transcription PCR (RT-PCR) was performed on the extracted RNA using the following primers: 5′ CAC TGC TTA AGC CTC AAT AAA GCT TGC C 3′ (HXB2 nucleotides 512–539) and 5′ TTT AAC CCT GCT GGG TGT GGT ATY CCT 3′ (HXB2 nucleotides 2851–2825) (Superscript III One-Step RT-PCR kit, Invitrogen, Carlsbad, CA). To enable recombination with an NL4-3 backbone, a second-round PCR was performed using 100-mer primers that were complementary to NL4-3 on either side of Gag-protease. A digested *gag-protease*-deleted pNL4-3 plasmid was then co-transfected with the patients’ *gag-protease* PCR amplicons into Tat-inducible green fluorescent protein (GFP) reporter GXR T cells by electroporation. The GXR T cells were cultured and monitored by flow cytometry for percentages of infected cells. Virus-containing culture supernatants were harvested and stored when approximately 30% of the cells became infected.

The titers of the harvested virus stocks were then assessed by determining the percentage of infected GXR T cells after 48 h of exposure, followed by calculating the amounts of virus stocks needed to achieve infection rates of 0.3%, i.e., a multiplicity of infection (MOI) of 0.003.

In the replication capacity assays, GXR T cells were cultured with virus stocks at an MOI of 0.003 for 6 days. The slope of the percentages of infected GXR T cells between day 3 and day 6 was calculated using the semilog method and then normalized by dividing by the slope of a wild-type NL4-3 virus included in the same assay.

### Statistical analyses

Comparisons of paired participants’ samples between different time points were assessed using the Wilcoxon matched-pairs signed-rank test. Nadir CD4^+^ T cell counts were determined as the nadir during the first 28 days after the detection of plasma viremia. Set point CD4^+^ T cell counts and set point viral loads were determined by calculating the averages between 28 days and 6 months after the detection of viremia. Relationships between cytokine levels, viral replicative capacity, CD4^+^ T cell parameters, CBC measurements, and viral loads were determined using Spearman’s rank-order correlation. Due to sample size limitations, corrections for multiple comparisons were not done. Multivariable linear regression analyses were conducted to assess the independent associations among some of the variables after adjusting for covariates. Since CBC measurements in the FRESH cohort were only available at the pre-infection and the earliest post-infection visits, we stratified the post-infection data according to the Fiebig stage at diagnosis (i.e., Fiebig stages I–II and Fiebig stages III–V) to determine the progressive effects of HIV during the acute phases of the infection. For comparison, we included CBC data from a chronic viremic HIV infection cohort. Cross-sectional comparisons between the pre-infection, Fiebig stages I–II, Fiebig stages III–V, and the chronic datasets were then done using the Wilcoxon rank-sum test (Mann-Whitney *U* test).

To simplify the visualization of the temporal variation in the cytokine profiles of the study participants, principal component analyses were done on the cytokines that showed elevation during acute HIV infection. The MetScape application was used within the Cytoscape platform to show the correlation network between different cytokines, immune cells, viral replication capacity, and other clinical laboratory measurements [30].

*P* values < 0.05 were considered statistically significant. All analyses were done on Stata version 15 (StataCorp) and GraphPad Prism version 7 (GraphPad Software, Inc).

## Results

### Characteristics of the study participants

Due to limitations in the availability of matched longitudinal plasma samples, 12 of the first 14 untreated participants for whom ART was deferred until immunological deterioration were assessed in the measurement of plasma cytokines levels. For comparison, 8 participants who received immediate ART during AHI were assessed to determine the impact of early ART. All but 1 participant were diagnosed at Fiebig stages I–II. One participant was diagnosed at Fiebig stage III. Notably, all participants, including the one diagnosed at Fiebig stage III, were on an upward plasma viral load trajectory at diagnosis. Early treatment was associated with the blunting of peak viremia and preservation of CD4^+^ T cell counts, consistent with our previous reports (Table 1 and Supplementary figure 1A-D) [15].

**Table 1:** Characteristics of study participants for whom cytokines were measured

	Acute untreated	Acute treated	*P* value^d^
Number of participants	12	8	
Female, *N* (%)	12 (100%)	8 (100%)	> 0.999
Age in years	21 (21–24)	22 (20–23)	0.917
Fiebig stages I–II at diagnosis, *N* (%)^a^	11 (92%)	8 (100%)	> 0.999
Fiebig stages I–II at ART initiation, *N* (%)	N/A	8 (100%)	N/A
Days from detection to ART initiation and range	N/A	1 (1–2)	N/A
Nadir CD4^+^ T cell counts^b^ (cells/μL)	329 (233–458)	840 (623–1008)	< 0.001
Peak viral load (log_10_ RNA copies/mL)	7.08 (6.93–7.75)	4.26 (3.45–5.13)	< 0.001
Days from detection to viral suppression and range	N/A	23 (7–63)	N/A
Set point CD4^+^ T cell counts (cells/μL)^c^	583 (555–630)	N/A	N/A
Set point viral load (log_10_ RNA copies/mL)^c^	4.74 (4.11–5.36)	N/A	N/A

Medians and interquartile ranges (in brackets) are shown for CD4^+^ T cell count and viral load measurements. Medians and ranges (in brackets) are shown for the number of days

*N/A* not applicable

^a^One participant in the acute untreated group was diagnosed at Fiebig stage III, but her viremia was still on an upward trajectory. At the time of diagnosis, her viral load was 5.94 log_10_ RNA copies/mL which rose to 6.51 log_10_ RNA copies/mL at the next study visit

^b^Nadir CD4^+^ T cell counts were determined as nadir during the first 28 days after the detection of plasma viremia

^c^Set point CD4^+^ T cell counts and set point viral loads were determined by calculating the averages between 28 days and 6 months after the detection of viremia in untreated individuals

^d^Statistical tests used: Fisher’s exact test was used for % female and Fiebig stage at diagnosis; all other comparisons were done using the Wilcoxon rank-sum test

### Acute HIV infection induces a cytokine/chemokine storm which is characterized by distinct kinetics and is abrogated by treatment at Fiebig stages I–II

**Fig. 1:**
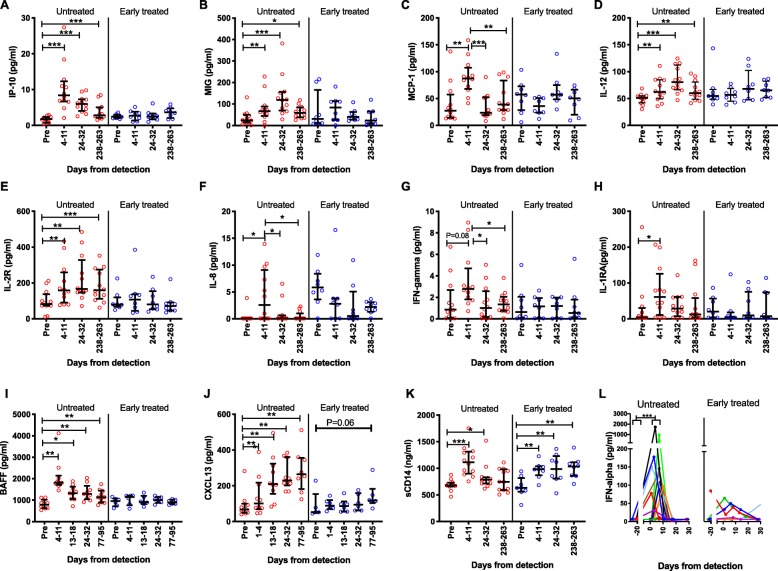
Untreated hyperacute HIV, but not ART early-treated hyperacute HIV, is associated with elevation of plasma cytokines that have distinct kinetics. **a** Interferon gamma-induced protein 10 (IP-10/CXCL-10). **b** Monokine induced by gamma interferon (MIG/CXCL-9). **c** Monocyte chemoattractant protein 1 (MCP-1). **d** Interleukin 12 (IL-12). **e** Soluble IL-2 receptor (IL-2R). **f** Interleukin 8 (IL-8). **g** Interferon gamma (IFN-gamma). **h** Interleukin-1 receptor antagonist (IL-1RA). **i** B cell-activating factor (BAFF/BLYS/TNFSF13B). **j** Chemokine (C-X-C motif) ligand 13 (CXCL13). **k** Soluble CD14. **l** Interferon alpha (IFN-alpha). *N* = 12 for untreated hyperacute HIV-infected participants (except CXCL13 and BAFF with *N* = 10). *N* = 8 for ART early-treated hyperacute HIV-infected individuals (except CXCL13 and BAFF with *N* = 6 and IFN-alpha with *N* = 7). Cytokine levels for one of the untreated participants were measured 434 days instead of 238–263 days after the detection of viremia. Each symbol represents an individual participant. Except for IFN-alpha, red symbols show the plasma levels in untreated participants and blue symbols show the plasma levels in ART early-treated participants. Horizontal lines and error bars in the scatter plots represent the median and interquartile range. In **l** (IFN-alpha), every colored line represents a participant. Statistical test used: Wilcoxon matched-pairs signed-rank test. *P* values < 0.05 were considered significant. *P < 0.05, **P < 0.01, ***P < 0.001. “Pre” refers to the pre-infection time point

In agreement with previous work, untreated AHI was associated with a surge in plasma cytokines, compared to matched pre-infection time points [1]. Of the 33 analytes measured, untreated individuals developed elevated levels of IP-10, MIG, MCP-1, IL-12, soluble IL-2 receptor, IL-8, IL-1RA, BAFF, CXCL13, IFN-alpha, and soluble CD14, and a trend in elevation of IFN-gamma in AHI (Fig. 1a–l). Notably, the kinetics of the markers during untreated infection differed between cytokines. MCP-1, IL-8, IFN-gamma, IL-1RA, and IFN-alpha had the fastest kinetics with a peak in the hyperacute phase and complete resolution after peak viremia (24–32 days after the detection of viremia) (Fig. 1c, f–h, l). Soluble CD14 had fast surge kinetics, but a complete resolution was only observed at the early chronic phase (238–263 days after the detection of viremia) (Fig. 1k). IP-10 and BAFF also displayed fast upward surge kinetics with a peak in the hyperacute phase (4–11 days after the detection of viremia) but only partial resolution after peak viremia (24–32 days after the detection of viremia) and later time points (Fig. 1a, i). On the other hand, MIG and IL-12 had slower kinetics with a peak after peak viremia (24–32 days after the detection of viremia) followed by partial resolution at the early chronic phase (238–263 days after the detection of viremia) (Fig. 1b, d). Soluble IL-2 receptor and CXCL13 had the slowest kinetics and did not show resolution even after peak viremia (Fig. 1e, j). Except for soluble CD14, there were no statistically significant elevations in plasma analytes among the early-treated participants, but we observed a non-significant trend when we compared CXCL13 levels pre-infection and at the early chronic phase (77–95 days after the detection of viremia) (*P* = 0.06). Thus, treating patients at Fiebig stages I–II abrogates the cytokine storm (Fig. 1a–l).

**Fig. 2:**
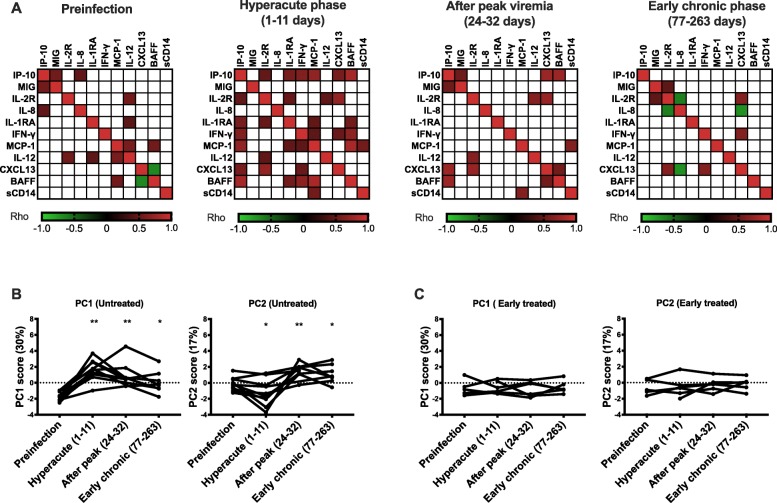
Hyperacute HIV is associated with multicollinearity among the elevated cytokines. **a** Correlation analyses for all participants at each time point among the cytokines that constituted the cytokine storm (except IFN-alpha) in hyperacute HIV infection, *N* = 20 (12 untreated hyperacute HIV-infected participants and 8 ART early-treated hyperacute HIV-infected individuals). The colors show Spearman’s rank-order correlation coefficients (rho) for the correlations that had *P* < 0.05. White squares indicate associations that were not statistically significant. **b** The kinetics of the first and second principal components (derived from cytokines that constituted the storm except for IFN-alpha which was detected at only one time point) among untreated participants. **c** The kinetics of the first and second principal components (derived from cytokines that constituted the storm except for IFN-alpha) among ART early-treated participants. Cytokine levels for one of the untreated participants were measured 434 days instead of 238–263 days after the detection of viremia. In **b** and **c**, every line represents a participant (untreated hyperacute HIV-infected participants, *N* = 10; ART early-treated hyperacute HIV-infected individuals, *N* = 6). Statistical test used in **b** and **c**: Wilcoxon matched-pairs signed-rank test. *P* values < 0.05 were considered significant. *P < 0.05, **P < 0.01, ***P < 0.001

Since inflammatory cytokines tend to be induced via related/overlapping pathways, we assessed whether there were statistical associations among the cytokines that were induced during hyperacute HIV infection. In correlation analyses on all individuals, there were significant positive correlations among many of the cytokines at different time points, confirming related/overlapping pathways (Fig. 2a). To summarize the dynamics of the cytokine responses, we conducted principal component analyses (PCA) on the cytokines and assessed the variation over time. Among untreated participants, pre-infection samples had the least variation, as depicted in the plots of the first and second principal components (i.e., PC1 and PC2). The highest variation was observed during the hyperacute phase, as depicted by the high dispersion (Fig. 2b). There were no differences in principal component scores between different time points in the early-treated participants, confirming that the cytokine profiles are relatively stable if patients are treated at Fiebig stages I–II (Fig. 2c).

### CXCL13 is positively associated with delayed suppression of viremia in early-treated individuals

**Fig. 3:**
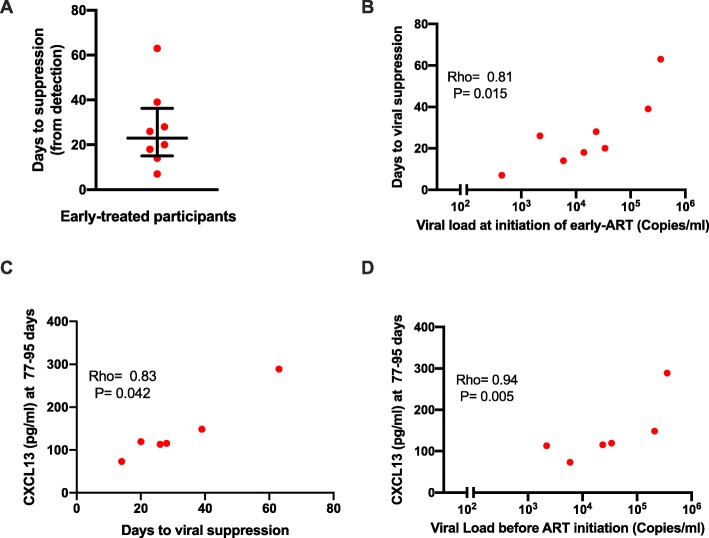
CXCL13 is positively associated with delayed suppression of viremia in early-treated individuals. **a** Duration to viral suppression in days among early-treated participants. **b** Correlation between duration to viral suppression in days and viral load at the time of initiating ART in early-treated individuals. **c** Correlation between duration to viral suppression and plasma CXCL13 levels at 3 months. **d** Correlation between viral load at the time of initiating ART and plasma CXCL13 levels at 3 months. Each symbol represents an individual participant (*N* = 6). Statistical test: Spearman’s rank-order correlation. *P* values < 0.05 were considered significant

Since control of viremia by ART largely abrogated the cytokine storm in the early-treated individuals, suggesting a role for persistent viremia in maintaining plasma cytokine levels, we checked if the duration to viral load suppression was associated with cytokine levels at early chronic time points (77–95 days for CXCL13 and BAFF or 238–263 days for the other cytokines) in the eight early-treated individuals. The median number of days to viral suppression was 23 (range 7–63 days) (Fig. 3a). Notably, there were positive correlations between the number of days to viral suppression and the viral loads at the time of initiation of ART (rho = 0.81, *P* = 0.015) (Fig. 3b). We observed positive correlations between days to viral suppression and plasma levels of CXCL13 at the early chronic phase (rho = 0.83, *P* = 0.042) (Fig. 3c). The plasma levels of CXCL13 at the early chronic stage were also positively correlated with the viral loads at the time of initiation ART (rho = 0.94, *P* = 0.005) (Fig. 3d), probably due to the positive association between viral loads at the time of initiation ART and the number of days to viral suppression. None of the other cytokine measurements at the early chronic phase correlated with the number of days to viral suppression. Thus, delayed suppression of viremia in early-treated individuals is associated with higher levels of plasma CXCL13 even after the suppression of viremia in the blood.

### HIV Gag-protease-driven replicative capacity is associated with the magnitude of inflammatory cytokines/chemokines in untreated hyperacute HIV infection

Since the hyperacute measurements best captured the highest variability in cytokine expression in untreated acute HIV infection, subsequent analyses for the relationships between cytokines and other parameters in untreated participants were performed using the cytokine responses during that phase.

Viral fitness has been proposed to determine the magnitude of the inflammatory response in early HIV infection [31]. Here, we assessed the correlation between the in vitro Gag-protease-driven replicative capacities of the transmitted/founder viruses with the levels of the hyperacute phase cytokines, viral loads, and CD4^+^ T cell counts in untreated acute HIV infection.

**Fig. 4:**
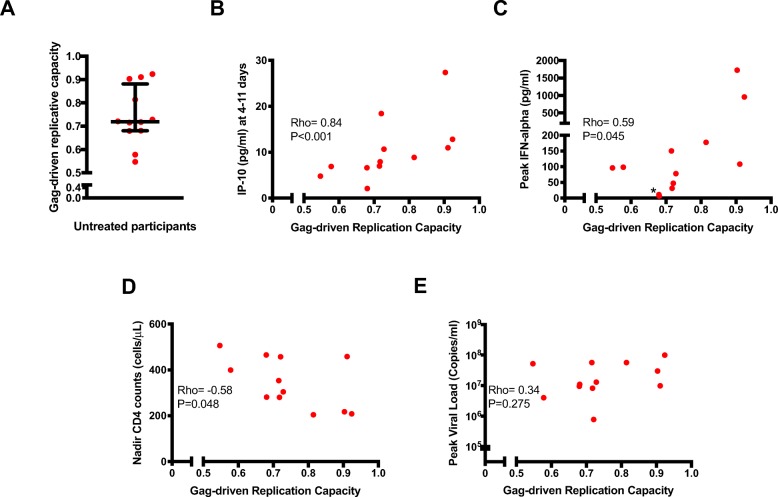
HIV Gag-driven replicative capacity predicts the magnitude of inflammatory cytokines and CD4^+^ T cell depletion in untreated hyperacute HIV infection. **a** Gag-driven replicative capacity among untreated participants. **b** Correlation between IP-10 in the hyperacute phase and replicative capacity. **c** Correlation between peak IFN-alpha and replicative capacity (* indicates two overlapping data points). **d** Correlation between nadir CD4^+^ T cell counts and replicative capacity. **e** Correlation between peak viral loads and replicative capacity. Each symbol represents an individual participant (*N* = 12). Statistical test: Spearman’s rank-order correlation. *P* values < 0.05 were considered significant

As expected, the transmitted/founder viruses among untreated participants differed among individuals with regard to Gag-protease-driven replicative capacities (Fig. 4a) and nucleotide sequences (Supplementary figure 2A-B) [29, 32]. Notably, most of the transmitted/founder viruses had attenuating polymorphisms that have been previously shown to reduce Gag-protease-driven replicative capacity [32]. Interestingly, we observed a trend of an inverse correlation between the total numbers of attenuating polymorphisms and Gag-protease-driven replicative capacities (rho = − 0.56, *P* = 0.067) (Supplementary figures 2C). We next investigated whether there were correlations between Gag-protease-driven replication capacities and cytokine levels in the untreated acutely infected individuals. Indeed, there was a strong positive correlation between Gag-protease-driven viral replicative capacity and IP-10 in the hyperacute phase (rho = 0.84, *P* < 0.001) and peak IFN-alpha (rho = 0.59, *P* = 0.045) (Fig. 4b, c). We also observed inverse correlations between Gag-protease-driven viral replicative capacity and nadir CD4^+^ T cell counts (rho = − 0.58, *P* = 0.048) (Fig. 4d). There were no correlations between Gag-protease-driven viral replicative capacity and peak viremia (Fig. 4e). In further multivariable linear regression analyses to determine the predictive effect of viral replicative capacity independent of viremia, the positive association between viral replicative capacities and levels of IP-10 during the hyperacute phase was observed even after adjusting for peak viremia. A similar but non-significant trend was observed between replicative capacities and levels of peak IFN-alpha in multivariable linear regression analyses after adjusting for peak viremia (Table 2). Thus, viruses with higher replication capacities induced a stronger IP-10 inflammatory response, independent of peak viremia.

**Table 2:** Linear regression analyses to determine the prediction of inflammatory cytokines and CD4^+^ T cell counts by Gag-protease-driven viral replicative capacity

Outcome variable	Predictor variable	Univariable analyses		Multivariable analyses	
		Coefficient (95% CI)	*P* value	Coefficient (95% CI)	*P* value
IP-10 at 1 week (log_10_ pg/mL)^a^	Replicative capacity	1.41 (0.13–2.69)	**0.034**	1.61 (0.28–2.95)	**0.023**
	Peak viral load^b^	− 0.04 (− 0.38–0.29)	0.777	− 0.14 (− 0.42–0.13)	0.274
Peak IFN-alpha (log_10_ pg/mL) ^a^	Replicative capacity	3.32 (0.12–6.53)	**0.044**	2.70 (− 0.52–5.93)	**0.091**
	Peak viral load^b^	0.60 (− 0.11–1.31)	0.088	0.43 (− 0.24–1.10)	0.176
Nadir CD4^+^ T cell counts (cells/μL)	Replicative capacity	− 483.67 (− 1013.23–45.88)	**0.069**	− 396.22 (− 945.81–153.37)	0.137
	Peak viral load^b^	− 85.69 (− 201–30.15)	0.130	− 61.41 (− 175.42–52.60)	0.254

Significant associations and non-significant trends are shown in bold

Number of participants in the analyses (*N*), 12

^a^Plasma cytokine levels were log_10_-transformed to attain normal distribution

^b^Peak viral load was expressed in log_10_ RNA copies/mL

### Levels of plasma cytokines/chemokines are associated with CD4^+^ T cell and viral load dynamics in untreated hyperacute HIV infection

**Fig. 5:**
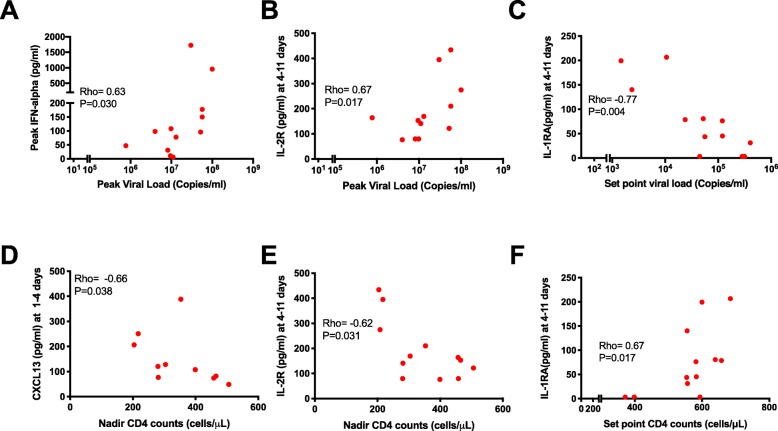
The magnitude of plasma cytokines predicts CD4^+^ T cell and viral load dynamics in untreated hyperacute HIV infection. **a** Correlation between peak IFN-alpha and peak viremia. **b** Correlation between hyperacute soluble IL-2 receptor and peak viremia. **c** Correlation between hyperacute IL-1RA and viral load set point. **d** Correlation between hyperacute CXCL13 and nadir CD4^+^ T cell counts. **e** Correlation between hyperacute soluble IL-2 receptor and nadir CD4^+^ T cell counts. **f** Correlation between hyperacute IL-1RA and set point CD4^+^ T cell counts. Each symbol represents an individual participant (*N* = 12 except CXCL13 with *N* = 10). Statistical test: Spearman’s rank-order correlation. *P* values < 0.05 were considered significant

The inflammatory cytokine responses in untreated early HIV could impact parameters such as peak viremia, set point viral loads, nadir CD4^+^ T cell counts, set point CD4^+^ T cell counts, and consequently disease progression. Among the untreated individuals, even though we did not observe an association between the nadir CD4^+^ T cell counts and the peak viremia, there was a notable inverse correlation between the set point viral loads and the set point CD4^+^ T cell counts (Supplementary figure 3A-B). In further correlation analyses using hyperacute plasma cytokines/chemokines in these untreated individuals, peak IFN-alpha showed a positive correlation with peak viremia (rho = 0.63, *P* = 0.030) (Fig. 5a). Soluble IL-2 receptor during the hyperacute phase (4–11 days) also correlated positively with peak viremia (rho = 0.67, *P* = 0.017) (Fig. 5b). These correlations of IFN-alpha and soluble IL-2 receptor against peak viremia suggest enhancement of viral replication by the inflammatory response, or conversely, higher viremia could be the driver of the observed inflammatory responses. IL-1RA at the peak acute stage correlated negatively with the later set point viral load in the early chronic phase (rho = − 0.77, *P* = 0.004), suggesting that its anti-inflammatory effects during untreated AHI could be associated with maintenance of useful immune responses that control viral replication later. CXCL13 had an inverse correlation with nadir CD4^+^ T cell counts, probably due to enhanced trafficking of CD4^+^ T cells away from the blood into lymphoid tissues and sites of inflammation (rho = − 0.66, *P* = 0.038) (Fig. 5d). Soluble IL-2 receptor correlated inversely with nadir CD4^+^ T cell counts (rho = − 0.62, *P* = 0.031) (Fig. 5e), suggesting a negative impact of inflammation by enhancing CD4^+^ T cell depletion, probably due to the enhanced viral replication, similar to previous reports [31]. In agreement with the inverse correlation between IL-1RA and set point viral load, there was a notable positive correlation between the analyte and set point CD4^+^ T cell counts (rho = 0.67, *P* = 0.017) (Fig. 5f).

### Acute HIV infection induces expansion of monocytes and depletion of lymphocytes, eosinophils, and basophils in the blood

CBC measurements were obtained prior to infection and at the first sampling visit after the detection of plasma viremia (before any participant was initiated on ART) in all participants. As such, all CBC determinations in the entire FRESH cohort were in the absence of ART and were therefore used to assess the earliest effects of untreated hyperacute HIV infection. To determine the kinetics of the respective immune cells in hyperacute HIV infection, we stratified the participants further according to the Fiebig stage at diagnosis, which was also the Fiebig stage at which post-infection CBC measurements were determined. For comparison, we used CBC data for viremic chronically infected HIV patients from a different cohort in the analyses (Table 3).

**Table 3:** Characteristics of participants involved in the determination of complete blood counts at initial detection of viremia

	Pre-infection	Fiebig stages I–II	Fiebig stages III–V	Chronic	*P* value^a^		
					Fiebig stages I–II	Fiebig stages III–V	Chronic
Number of participants	70	60	15	33	N/A	N/A	N/A
Female, *N* (%)	70 (100%)	60 (100%)	15 (100%)	32 (97%)	1.000	1.000	0.143
Age in years	21.1 (19.8–22.1)	21.2 (19.9–22.5)	22.5 (20.8–23.7)	22.9 (21.4–24.3)	0.508	0.039	< 0.001
Viral load (log10 copies/mL)	N/A	4.20 (3.50–4.92)	6.26 (4.88–7.20)	3.60 (3.04–4.31)	N/A	N/A	N/A
CD4^+^ T cell counts (cells/μL)	891 (751–1047)	763 (538–911)	444 (367–553)	599 (470–756)	< 0.001	< 0.001	< 0.001

Values for age, viral load, and CD4^+^ T cell counts are medians and interquartile ranges

^a^Statistical test used: Wilcoxon rank-sum test (Mann-Whitney *U* test) was used to compare the differences in age and CD4+ T cell counts between pre-infection and post-infection time points. Chi-square test was used to assess the differences in gender distribution

**Fig. 6:**
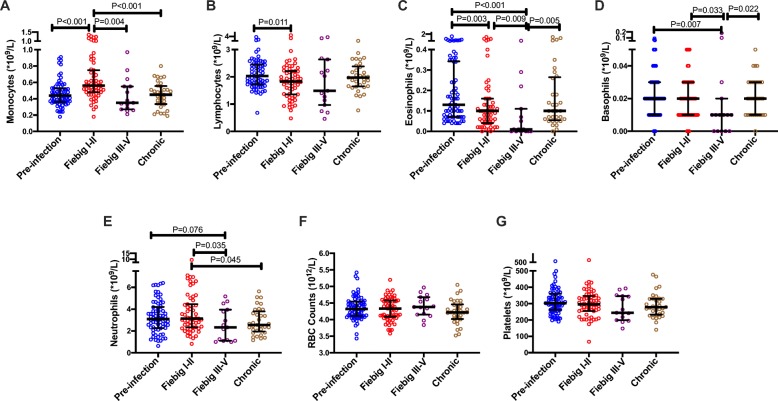
Hyperacute HIV infection is associated with dysregulation of blood lymphoid and myeloid cells. **a** Monocyte absolute counts. **b** Total lymphocyte absolute counts. **c** Eosinophil absolute counts. **d** Basophil absolute counts. **e** Neutrophil absolute counts. **f** Red cell counts. **g** Platelet counts. For all cellular components, measurements before HIV infection (blue symbols, *N* = 70), in AHI at Fiebig stages I–II (red symbols, *N* = 60) and in AHI at Fiebig stages III–V (purple symbols, *N* = 15) from the FRESH acute infection cohort are shown. Measurements from a different chronic cohort (brown symbols, *N* = 33) are included for comparison purposes. Each symbol represents an individual participant. Horizontal lines and error bars in the scatter plots represent the median and interquartile range. Statistical test used: Wilcoxon rank-sum test (Mann-Whitney *U* test). *P* values < 0.05 were considered significant

The effects of hyperacute HIV on different compartments of immune cells differed in their directionality and kinetics. Untreated Fiebig stages I–II AHI was associated with an expansion in the absolute numbers of blood monocytes (*P* < 0.001). Interestingly, blood monocyte numbers in Fiebig stages III–V and chronically infected HIV patients were comparable to the pre-infection values, suggesting that the expansion of monocytes in acute HIV is transient and resolves as patients progress beyond Fiebig stages I–II (Fig. 6a). We observed a reduction of absolute numbers of blood lymphocytes in untreated Fiebig stages I–II AHI (*P* = 0.011), probably due to the depletion of CD4^+^ T cells, natural killer cells, and B cells as previously reported [14, 15]. There were no significant differences between pre-infection FRESH participants and chronic patients with regard to total lymphocytes, probably due to the partial recovery of CD4^+^ T cell counts and expansion of activated CD8 T cells as the infection progresses (Fig. 6b). Untreated acute HIV was also associated with a progressive reduction in absolute numbers of blood eosinophils at Fiebig stages I–II and III–V (*P* = 0.003 and *P* < 0.001, respectively). However, there were no differences in numbers of blood eosinophils between chronically infected participants and pre-infection time points, suggesting recovery as patients moved into the chronic phase of infection (Fig. 6c). There was a reduction in absolute numbers of blood basophils in patients who were diagnosed at Fiebig stages III–V (*P* = 0.007). Similar to the eosinophils, the numbers of basophils in chronically infected participants were comparable to those at pre-infection time points, suggesting recovery in chronic infection (Fig. 6d). We did not observe any significant changes in numbers of blood neutrophils when we compared post-infection time points with the pre-infection time point. However, comparisons between Fiebig stages I–II and later stages suggested a depletion of neutrophils with disease progression at Fiebig stages III–V (*P* = 0.035) and chronic infection (*P* = 0.045) (Fig. 6e). We did not observe changes in the absolute numbers of red blood cells and platelets (Fig. 6f, g). All these trends remained similar when we assessed the differences using the percentages of the immune cells (Supplementary figure 4A-E). Considering that our measurements were only done at the earliest sampling time point, which was also before the initiation of ART, we did not explore the possible HIV-induced changes in cellular populations at subsequent time points in untreated acute HIV infection and the effects of early ART.

### Plasma cytokines/chemokines are associated with reduced blood counts of lymphocytes, eosinophils, and basophils in untreated acutely HIV-infected patients

Both myeloid and lymphoid blood cells have been implicated in the secretion of inflammatory cytokines/chemokines in HIV. HIV-induced cytokines/chemokines could also in turn modulate the generation, maintenance, and trafficking of the different lineages of blood cells. We therefore assessed if there were statistically significant associations between the immune cells and plasma cytokines/chemokines during the hyperacute phase in the untreated participants.

**Fig. 7:**
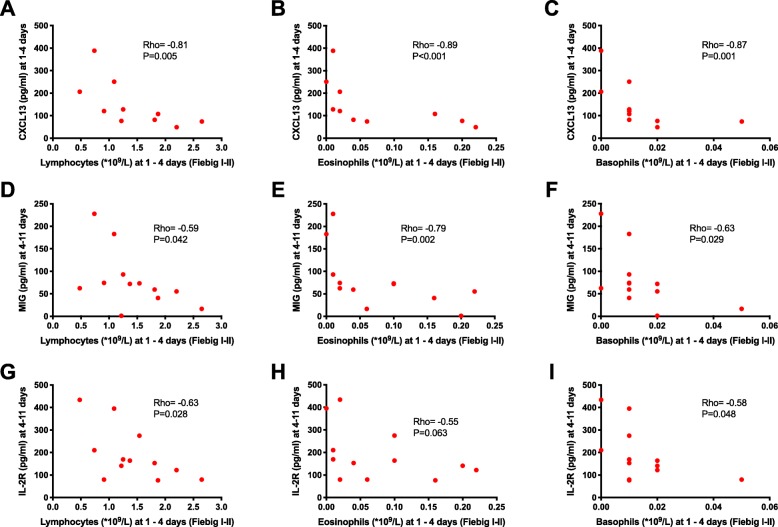
Plasma cytokines/chemokines are associated with reduced blood counts of lymphocytes, eosinophils, and basophils in untreated acutely HIV-infected patients. **a** Correlation between CXCL13 and total lymphocytes. **b** Correlation between CXCL13 and eosinophils. **c** Correlation between CXCL13 and basophils. **d** Correlation between MIG/CXCL9 and total lymphocytes. **e** Correlation between MIG/CXCL9 and eosinophils. **f** Correlation between MIG/CXCL9 and basophils. **g** Correlation between soluble IL-2 receptor and total lymphocytes. **h** Correlation between soluble IL-2 receptor and eosinophils. **i** Correlation between soluble IL-2 receptor and basophils. The measurements of cytokines and blood cell counts were in the hyperacute phase of HIV infection. Each symbol represents an individual participant (*N* = 12 except CXCL13 (**a**–**c**) with *N* = 10). Statistical test: Spearman’s rank-order correlation. *P* values < 0.05 were considered significant

There were no associations between the hyperacute cytokine/chemokine measurements and monocyte counts in untreated acute HIV infection. However, there were notable inverse correlations between the chemokines CXCL13 and total lymphocytes (rho = − 0.81, *P* = 0.005) (Fig. 7a), CXCL13 and eosinophils (rho = − 0.89, *P* < 0.001) (Fig. 7b), CXCL13 and basophils (rho = − 0.87, *P* = 0.001) (Fig. 7c), MIG and lymphocytes (rho = − 0.59, *P* = 0.042) (Fig. 7d), MIG and eosinophils (rho = − 0.79, *P* = 0.002) (Fig. 7e), and MIG and basophils (rho = − 0.63, *P* = 0.029) (Fig. 7f). Notably, there were no significant associations between peak viremia and any of the immune cells. However, since viremia would be expected to contribute to the dysregulation of the immune cells in AHI, we conducted additional multivariable linear regression analyses to assess the predictive effect of the cytokines on the immune cells after adjusting for peak viremia. The associations between CXCL13 and total lymphocytes, CXCL13 and eosinophils, and MIG and eosinophils were maintained even after adjusting for peak viremia (Supplementary table 1). Considering that CXCL13 and MIG are chemokines that mediate trafficking of immune cells away from the blood into the lymphoid tissues and sites of inflammation, our observations suggest chemokine-mediated sequestration of lymphocytes, eosinophils, and basophils in tissues. We also observed an inverse correlation between soluble IL-2 receptor and lymphocytes (rho = − 0.63, *P* = 0.028) (Fig. 7g), a non-significant trend of an inverse correlation between soluble IL-2 receptor and eosinophils (rho = − 0.55, *P* = 0.063) (Fig. 6h), and an inverse correlation between soluble IL-2 receptor and basophils (rho = − 0.58, *P* = 0.048) (Fig. 7i). In multivariable linear regression analyses, we observed a non-significant trend of negative prediction of lymphocytes by soluble IL-2 receptor after adjusting for peak viremia (Supplementary table 1). Even though we did not observe significant changes in platelets in hyperacute HIV infection, there was a positive correlation between post-infection platelet counts and interferon gamma during the hyperacute phase (rho = 0.62, *P* = 0.024) (Supplementary figure 5A). Likewise, even though there were no significant changes in neutrophils when we compared the pre-infection time point to AHI time points, we observed positive correlations between hyperacute neutrophil counts and IP-10 (rho = 0.74, *P* = 0.006) (Supplementary figure 5B), neutrophils and IL-8 (rho = 0.58, P = 0.048) (Supplementary figure 5C), and neutrophils and BAFF (rho = 0.67, *P* = 0.033) (Supplementary figure 5D), suggesting that neutrophils could be secreting some of the cytokines.

### Acute HIV infection induces a complex network of associations between cytokines, immune cells, clinical parameters, and viral replication capacity

**Fig. 8:**
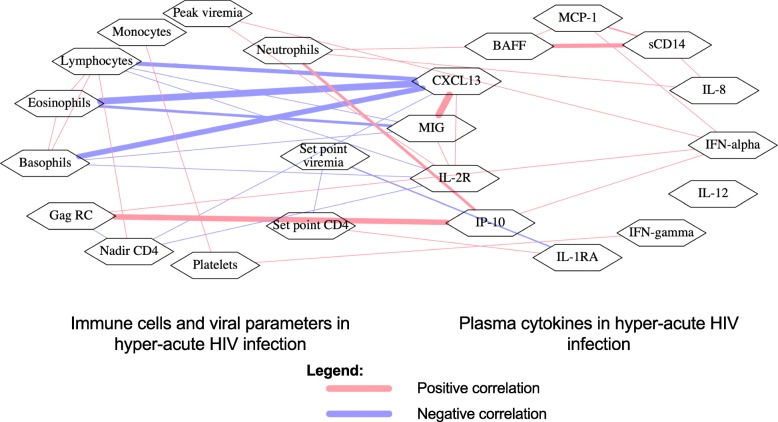
Correlation network showing a summary of the relationships between cytokines, CD4^+^ T cell dynamics, viral load dynamics, Gag-driven viral replication capacity, and hematological parameters in untreated hyperacute HIV infection. Statistical test used: Spearman’s rank-order correlation. Red lines show significant positive correlations. Blue lines show significant inverse correlations. The width of the line indicates the strength of Spearman’s correlation coefficient (rho). Only correlations that have *P* < 0.05 are shown. Gag RC, Gag-driven viral replication capacity (*N* = 12 except CXCL13 and BAFF with *N* = 10)

Untreated AHI was associated with complex relationships between plasma cytokines, immune cell dynamics, viral load dynamics, and viral replicative capacities (Fig. 8). The inverse correlations of CXCL13 with eosinophils, basophils, lymphocytes, and nadir CD4^+^ T cells were particularly interesting and suggested a role for CXCL13 in the sequestration of the immune cells in tissues during AHI. We observed similar associations with MIG, another chemokine that mediates trafficking to inflamed tissues, and soluble IL-2 receptor, a marker of inflammation. We also observed some positive associations among some of the cytokines, suggesting overlapping pathways leading to their induction. There were some positive associations among the immune cells, such as the positive correlations between lymphocytes, eosinophils, and basophils, suggesting common drivers of the depletions of those immune cells in AHI. Viral replication capacity correlated positively with IP-10 and IFN-alpha, and inversely with nadir CD4^+^ T cell counts, indicating the contribution of viral virulence on immunological deterioration and inflammation in AHI. Positive correlations of neutrophils against IL-8, BAFF, and IP-10 indicated a possibility of neutrophils being a source of some of the cytokines in AHI.

## Discussion

In this study, we assessed the magnitude and nature of the cytokine storm in untreated and early-treated hyperacute HIV-1 subtype C infection and determined the cytokine associations with viral and immunological profiles. In agreement with previous reports from settings where other HIV-1 subtypes dominate, we observed a cytokine/inflammatory storm [1, 7]. In particular, we observed an elevation of IP-10, MIG, IFN-gamma, IFN-alpha, MCP-1, IL-12, soluble IL-2 receptor, IL-1RA, IL-8, BAFF, CXCL13, and soluble CD14 in untreated individuals. In contrast, only plasma soluble CD14 was significantly elevated in the early-treated participants. Notably, the cytokines differed in their plasma kinetics, possibly because of the differences in activation kinetics of secreting cells, variations in transcriptional control, and/or differences in half-lives of the cytokines. We also report previously undescribed depletion of eosinophils, depletion of basophils, and a transient expansion of monocytes in AHI, thus providing new insights on the pathogenesis of AHI. AHI was also associated with a modest depletion of lymphocytes, probably due to the depletions of CD4^+^ T cells, natural killer cells, and B cells that have been reported previously [14, 15]. Thus, our study in young women who are infected with HIV-1 subtype C supports the concept of the cytokine/inflammatory storm in untreated AHI that has been observed in other cohorts. However, we could not do direct comparisons for intersubtype cytokine storm differences because our cohort was exclusively infected with only subtype C, and comparison with other cohorts would be confounded by sex, age, ethnic background, and methodological approaches for measuring cytokines/chemokines.

HIV-induced inflammation has been shown to affect a wide range of cellular compartments and tissues. For example, the elevated risks of developing cardiovascular complications and kidney disease in HIV patients have been linked to the HIV-induced inflammatory response in both untreated and ART-treated HIV patients [20–22, 33, 34]. Furthermore, the inflammatory response contributes to immune deterioration by mediating CD4^+^ T cell depletion and enhancing viral transcription [17–19]. HIV virulence and viral load dynamics have in turn been implicated in modulating the magnitudes and kinetics of inflammatory cytokines [7, 31]. In addition, some cellular compartments have been shown to be sources or targets of the HIV-induced cytokines [35–37]. By assessing the relationship between the cytokines during untreated hyperacute HIV infection and viral fitness, viral load dynamics, CD4^+^ T cell dynamics, and immune cell dynamics, we shed light on the effects of the cytokine storm on various cells and/or tissues.

Our observation on plasma soluble CD14 was unexpected in that in the untreated individuals, there was a surge in plasma soluble CD14 at the peak acute stage, followed by a spontaneous resolution in the early chronic stage. On the other hand, the early-treated participants had a modest but sustained elevation of the analyte. A similarly unexpected effect of ART on plasma soluble CD14 has been reported in chronically infected individuals whereby patients with high CD4^+^ T cell counts experienced further elevation of soluble CD14 upon treatment, unlike patients with low CD4 counts who experienced a reduction of the analyte [38]. Thus, the biology of soluble CD14 could be complicated by interactions between the inflammatory response, disease progression, and overall immune status. The trends observed in our study need further mechanistic investigations.

We also observed interesting correlations between CXCL13 levels at late time points and durations to viral suppression in early-treated individuals. Plasma CXCL13 has been reported to be a marker of germinal center activity and predicts the development of cross-reactive anti-HIV-neutralizing antibodies [6, 39]. On the other hand, viral replication, even in ART-treated individuals, has been shown to majorly occur in follicular helper T cells in germinal centers [40, 41]. Considering that occult viral replication could persist in B cell follicles of early-treated individuals, especially in those who take long to attain viral suppression in the blood, plasma CXCL13 after viral suppression could be a marker of continued germinal center activity that maintains the viral replication in lymphoid tissues of treated patients, a phenomenon that needs further investigation.

In agreement with a previous study that reported a relationship between viral replicative fitness and the magnitude of the inflammatory response in untreated AHI, we observed positive correlations between Gag-protease-driven replicative capacity and the cytokines IP-10 and IFN-alpha [31]. Other studies have reported faster CD4^+^ T cell decline in patients who are infected with faster-replicating viruses, an effect that could be driven by the strong inflammatory response induced by the fitter viruses [31, 32]. Thus, modulating inflammatory cytokines could reduce CD4^+^ T cell depletion in AHI with possible benefits as the patients progress into the chronic phase.

In the assessment of the relationships between cytokines and immune cell compartments in untreated individuals, the chemokine CXCL13 correlated inversely with lymphocytes, eosinophils, and basophils, suggesting chemokine-induced sequestration of the cells in tissues. CXCL13 is associated with the trafficking of cells into B cell follicles [6, 39]. Lymphocytes such as B cells and follicular T cells express CXCR5, the receptor for CXCL13, and HIV could mediate their sequestration in the lymph nodes via the release of CXCL13 [6, 39]. Indeed, AHI is associated with lymphadenopathy due to the accumulation of lymphocytes in the lymph nodes [42]. Even though the inverse correlations between CXCL13 and eosinophils and basophils were surprising, tissue eosinophils have been shown to produce CXCL13 or induce its expression in macrophages during inflammation in mice [43, 44], suggesting that the sequestration of eosinophils in tissues could promote the production of CXCL13 in tissue macrophages during hyperacute HIV infection. It will be important to determine if HIV also induces CXCR5 expression in eosinophils and basophils to render them responsive to CXCL13, leading to trafficking away from the blood compartment. The observations of similar inverse correlations between the chemokine MIG/CXCL9 and eosinophils, basophils, and lymphocytes suggest that the trafficking of these cells into inflamed tissues could also occur as a result of signaling via CXCR3, the chemokine receptor for MIG/CXCL9. The expression of CXCR3 has been described in lymphocytes and eosinophils, but further studies are needed to determine the occurrence and immunological implications of any CXCR3-dependent trafficking of these cells in hyperacute HIV infections [45]. The inverse correlation of lymphocytes, eosinophils, and basophils with soluble IL-2 receptor also suggests possible depletion due to the generalized inflammatory response. Increased HIV-induced death of lymphocytes by apoptosis and pyroptosis has been reported previously [46–48]. As such, HIV-induced increased cell death of eosinophils and basophils in hyperacute HIV infection cannot be ruled out. Considering that very little is known on the role of eosinophils and basophils in HIV pathogenesis, further mechanistic studies could reveal novel targets for intervention in treatment strategies to improve the quality of life of HIV-infected persons. For instance, eosinophils have been shown to promote IgA responses in the gut in a mouse model [49]. Considering that IgA responses were associated with reduced antibody-mediated protection in the RV144 vaccine trial, it will be important to determine if eosinophils home to the gut to promote unfavorable IgA responses in AHI [50].

We observed positive correlations between neutrophil counts and some of the cytokines, namely IP-10, IL-8, and BAFF, suggesting that neutrophils could significantly contribute to the cytokine storm. Neutrophils are the most abundant immune cells in the blood and can secrete these cytokines in inflammatory conditions [51]. Due to their large numbers, even subtle changes that do not translate to statistically significant quantitative differences in neutrophils, as well as hidden qualitative changes, could significantly impact the cytokine environment in hyperacute HIV. Additional mechanistic studies are needed to characterize the role of neutrophils in acute HIV.

The longitudinal nature of this study, with sampling performed prior to and at regular intervals immediately following HIV infection, enabled us to reliably determine the temporal changes and effects of plasma cytokines/chemokines during untreated and early-treated hyperacute HIV infection. Due to this multidimensional approach, we also provide a comprehensive picture of the associations that define AHI with regard to cytokine levels, viral load, CD4^+^ T cells, and immune cell parameters. The logistical difficulties of obtaining appropriate samples could explain the paucity of similar studies on hyperacute HIV-infected patients. The main limitation of the study is the fact that we had few participants for the cytokine measurements and our study might have been underpowered to detect some changes and associations. In addition, even though we observed depletions of lymphocytes, eosinophils, and basophils during hyperacute HIV infection before ART, we did not have CBC measurements after the initiation of ART in the early-treated individuals. Thus, we could not determine if those cellular defects persisted or were reversed by early ART. Nevertheless, we reveal several novel pathways that need further investigations to determine the consequences of the HIV-induced immune changes in the acute phase of infection.

## Conclusions

Untreated AHI caused a cytokine storm, depletion of lymphocytes, depletion of eosinophils, depletion of basophils, and a transient increase of monocytes in the peripheral blood. The Gag-protease-driven replicative capacity of HIV-1 was positively associated with the magnitude of some of the cytokines that constitute the cytokine storm, especially IP-10, suggesting a role for viral virulence in driving the inflammatory response. Chemokines CXCL13 and MIG were inversely associated with numbers of lymphocytes, eosinophils, and basophils in the blood, suggesting a role for sequestration of the immune cells in tissues in AHI. Thus, complex interactions between plasma cytokines, immune cells, viral factors, and clinical parameters characterize AHI.

## Supplementary information

**Additional file 1:** Supplementary table 1. Linear regression analyses to determine the prediction of immune cells numbers by plasma cytokines after adjusting for peak viremia

**Additional file 2: **Supplementary figure 1. Viral load and CD4^+^ T cell kinetics in participants for whom cytokines were measured. A. Viral loads kinetics in untreated participants (*N* = 12). B. Viral load kinetics in ART early-treated participants (*N* = 8). C. CD4^+^ T cell kinetics in untreated participants (*N* = 12). D. CD4^+^ T cell kinetics in ART early-treated participants (*N* = 8). Each line represents an individual participant. Arrows indicate time points that were used for determination of plasma cytokines by Luminex.

**Additional file 3: **Supplementary figure 2. Presence of attenuating polymorphisms predicts replication capacity of transmitted/founder viruses. A. Similarity of transmitted/founder viruses to the consensus sequence. B. Phylogenetic tree of the transmitted/founder viruses. Viruses with high replication capacity (above the median value) are shown in red. C. Correlation between the number of attenuating polymorphisms and replication capacity. Statistical test: Spearman’s rank-order correlation. *P* values < 0.05 were considered significant.

**Additional file 4: **Supplementary figure 3. Set point viral loads are inversely associated with set point CD4^+^ T cell counts in untreated individuals. A. Correlation between peak viral loads and nadir CD4^+^ T cell counts. B. Correlation between set point viral loads and set point CD4^+^ T cell counts. Every symbol represents a participant (*N* = 12). Statistical test: Spearman’s rank-order correlation. *P* values < 0.05 were considered significant.

**Additional file 5: **Supplementary figure 4. Hyperacute HIV infection is associated with dysregulation of proportions of blood lymphoid and myeloid cells. A. Monocytes percentages. B. Total lymphocytes percentages. C. Eosinophils percentages. D. Basophils percentages. E. Neutrophils percentages. For all cellular components, measurements before HIV infection (blue symbols, *N* = 70), in AHI at Fiebig stage I-II (red symbols, *N* = 60) and in AHI at Fiebig stage III-V (purple symbols, *N* = 15) from the FRESH acute infection cohort are shown. Measurements from a different chronic cohort (brown symbols, *N* = 33) are included for comparison purposes. Each symbol represents an individual participant. Horizontal lines and error bars in scatter plots represent median and interquartile range. Statistical tests used: Wilcoxon rank-sum test (Mann-Whitney U test). *P* values < 0.05 were considered significant.

**Additional file 6: **Supplementary figure 5. Hyperacute plasma cytokines/chemokines are associated with hematological dysregulations. A. Correlation between interferon gamma and platelets. B. Correlation between IP-10/CXCL10 and neutrophils. C. Correlation between IL-8 and neutrophils. D, Correlation between BAFF and neutrophils. The measurements of cytokines and blood cells counts were in the hyperacute phase of HIV infection. Every symbol represents a participant (*N* = 12 except BAFF with *N* = 10). Statistical test: Spearman’s rank-order correlation. *P* values < 0.05 were considered significant.

## Data Availability

The datasets used and/or analyzed during the current study are available from the corresponding author on request.
