# Supplementary material for: Association between the cytokine storm, immune cell dynamics, and viral replicative capacity in hyperacute HIV infection
Source: BMC Med. 2020 Mar 25;18:81. doi: 10.1186/s12916-020-01529-6 (PMC7093991; doi:10.1186/s12916-020-01529-6)
Supplement: Supplementary file 1 — Additional file 1: Supplementary table 1. Linear regression analyses to determine the prediction of immune cells numbers by plasma cytokines after adjusting for peak viremia [file 12916_2020_1529_MOESM1_ESM.docx]

**Supplementary table 1: Linear regression analyses to determine the prediction of immune cells numbers by plasma cytokines after adjusting for peak viremia**

| **Outcome variable** | **Predictor variable** | **Univariable analyses** | | **Multivariable analyses*** | |
| --- | --- | --- | --- | --- | --- |
|  |  | **Coefficient**  **(95% CI)** | **P value** | **Coefficient**  **(95% CI)** | **P value** |
| Lymphocytes counts at 1-4 days (*10^9^/L) | CXCL13 at 1-4 days  (log_10_ pg/ml) | **-1.89**  **(-3.22 – -0.55)** | **0.012** | **-1.79**  **(-3.40 – -0.19)** | **0.034** |
|  | MIG at 4-11 days  (log_10_ pg/ml) | -0.28  (-0.99 – 0.43) | 0.405 | -0.24  (-1.02 – 0.53) | 0.496 |
|  | IL-2R at 4-11 days  (log_10_ pg/ml) | **-1.47**  **(-2.85 – -0.08)** | **0.040** | **-1.66**  **(-3.38 – 0.06)** | **0.056** |
|  | Peak viral load  (log_10_ RNA copies/ml) | -0.22  (-0.94 – 0.51) | 0.519 |  |  |
| Eosinophils counts at 1-4 days (*10^9^/L) | CXCL13 at 1-4 days  (log_10_ pg/ml) | **-0.22**  **(-0.40 – -0.04)** | **0.024** | **-0.24**  **(-0.45 – -0.03)** | **0.033** |
|  | MIG at 4-11 days  (log_10_ pg/ml) | **-0.08**  **(-0.15 – -0.01)** | **0.034** | **-0.08**  **(-0.16 – -0.00)** | **0.048** |
|  | IL-2R at 4-11 days  (log_10_ pg/ml) | -0.12  (-0.32 – 0.08) | 0.202 | -0.14  (-0.39 – 0.11) | 0.233 |
|  | Peak viral load  (log_10_ RNA copies/ml) | -0.02  (-0.11 – 0.08) | 0.700 |  |  |
| Basophils counts at 1-4 days (*10^9^/L) | CXCL13 at 1-4 days  (log_10_ pg/ml) | **-0.03**  **(-0.07 – 0.00)** | **0.056** | -0.03  (-0.07 – 0.01) | 0.115 |
|  | MIG at 4-11 days  (log_10_ pg/ml) | -0.01  (-0.02 – 0.00) | 0.115 | -0.01  (-0.02 – 0.00) | 0.173 |
|  | IL-2R at 4-11 days  (log_10_ pg/ml) | **-0.03**  **(-0.06 – 0.00)** | **0.074** | -0.03  (-0.06 – 0.01) | 0.166 |
|  | Peak viral load  (log_10_ RNA copies/ml) | -0.01  (-0.02 – 0.01) | 0.296 |  |  |

*the predictive effects of cytokines on immune cells numbers were determined in multivariable analyses after adjusting for peak viremia.
