# Supplementary figures and images for: Association between the cytokine storm, immune cell dynamics, and viral replicative capacity in hyperacute HIV infection
Source: BMC Med. 2020 Mar 25;18:81. doi: 10.1186/s12916-020-01529-6 (PMC7093991; doi:10.1186/s12916-020-01529-6)

Supplementary figure 1

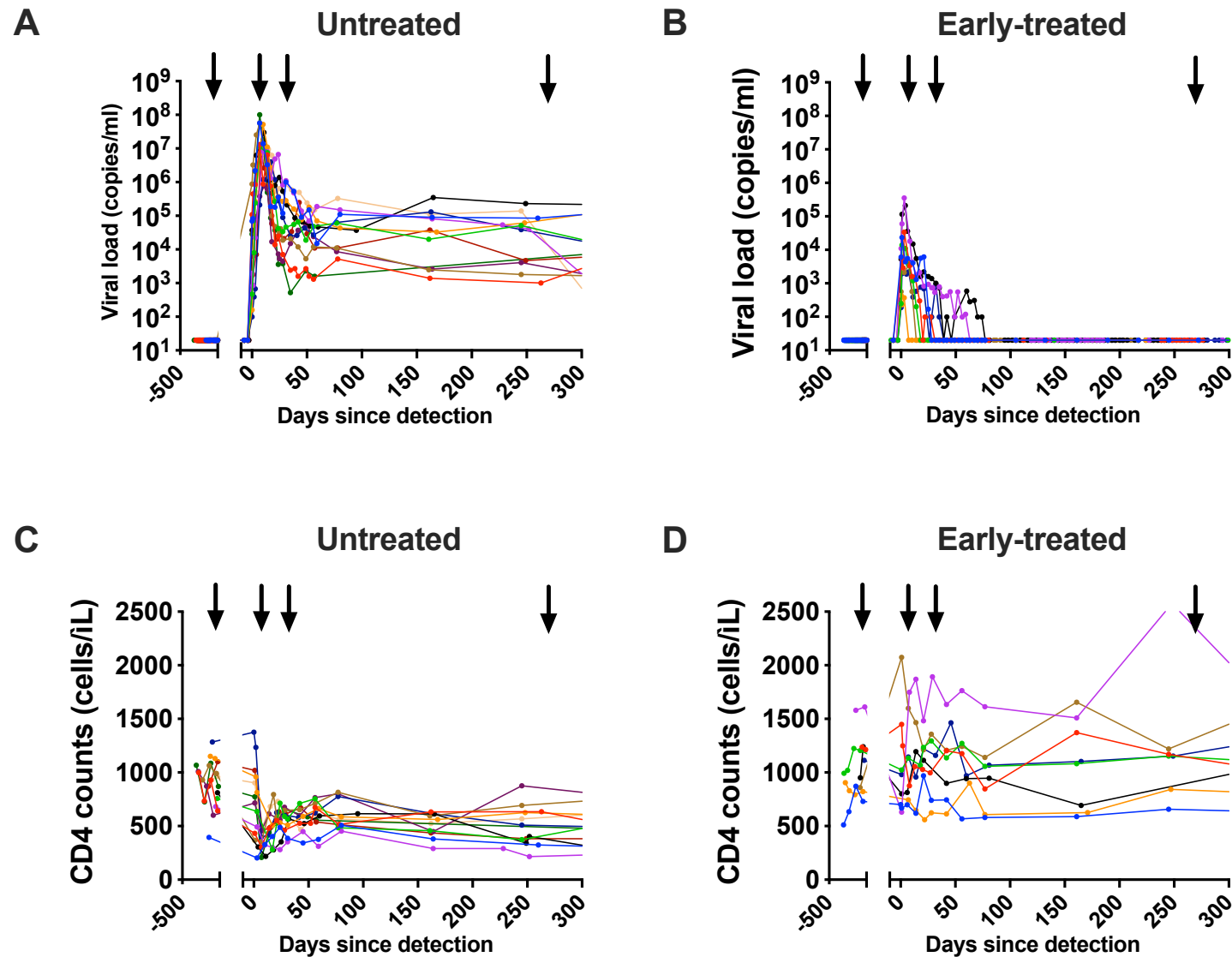

Supplement: Supplementary file 2 — Additional file 2: Supplementary figure 1. Viral load and CD4+ T cell kinetics in participants for whom cytokines were measured. A. Viral loads kinetics in untreated participants (N = 12). B. Viral load kinetics in ART early-treated participants (N = 8). C. CD4+ T cell kinetics in untreated participants (N = 12). D. CD4+ T cell kinetics in ART early-treated participants (N = 8). Each line represents an individual participant. Arrows indicate time points that were used for determination of plasma cytokines by Luminex. [file 12916_2020_1529_MOESM2_ESM.pdf]

Supplementary figure 2

A

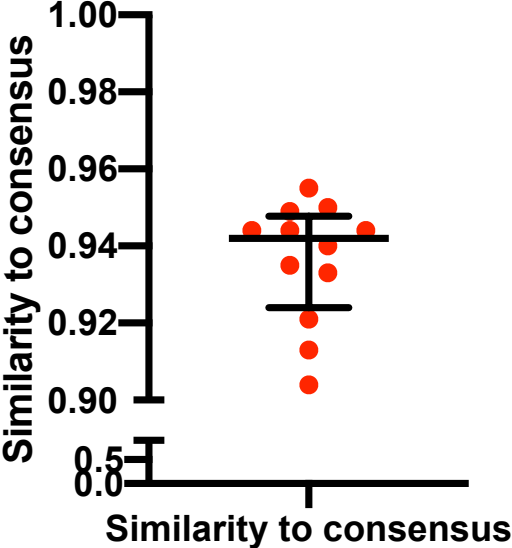

B

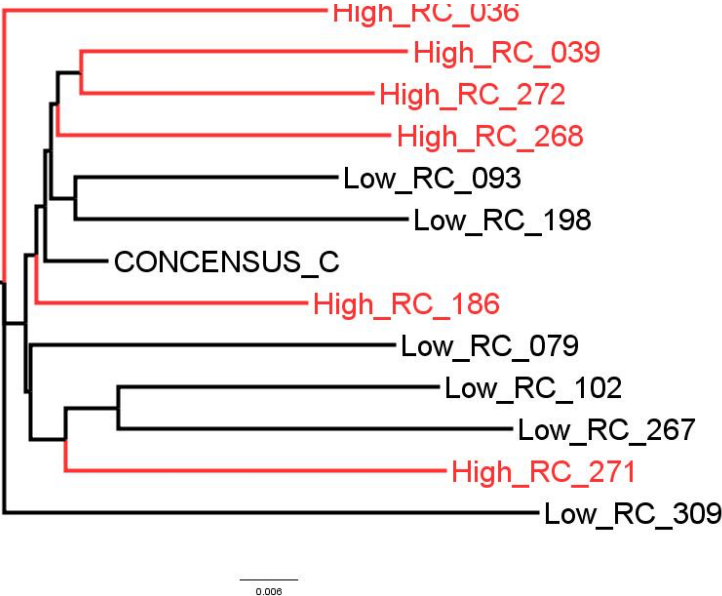

C

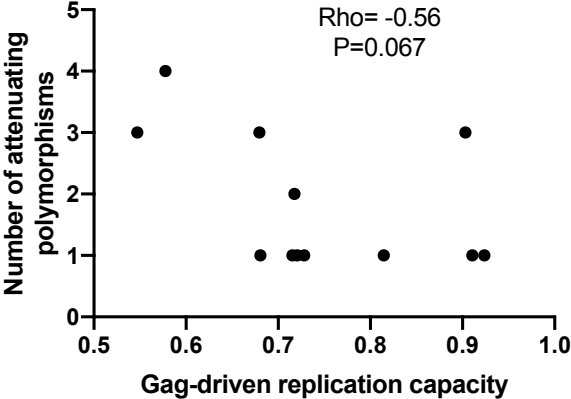

Supplement: Supplementary file 3 — Additional file 3: Supplementary figure 2. Presence of attenuating polymorphisms predicts replication capacity of transmitted/founder viruses. A. Similarity of transmitted/founder viruses to the consensus sequence. B. Phylogenetic tree of the transmitted/founder viruses. Viruses with high replication capacity (above the median value) are shown in red. C. Correlation between the number of attenuating polymorphisms and replication capacity. Statistical test: Spearman’s rank-order correlation. P values < 0.05 were considered significant. [file 12916_2020_1529_MOESM3_ESM.pdf]

Supplementary figure 3

A

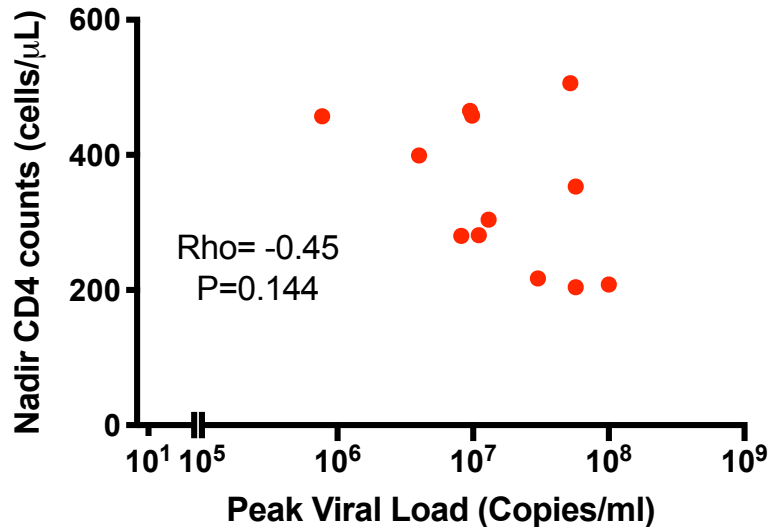

B

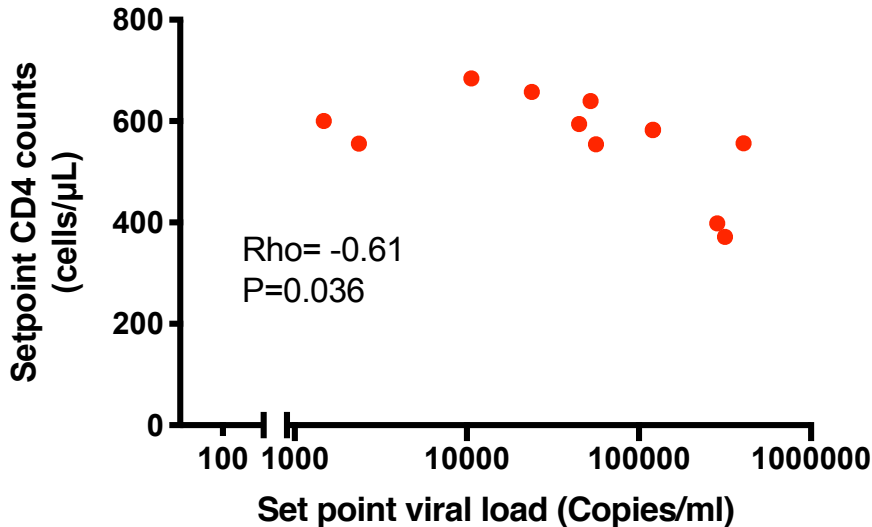

Supplement: Supplementary file 4 — Additional file 4: Supplementary figure 3. Set point viral loads are inversely associated with set point CD4+ T cell counts in untreated individuals. A. Correlation between peak viral loads and nadir CD4+ T cell counts. B. Correlation between set point viral loads and set point CD4+ T cell counts. Every symbol represents a participant (N = 12). Statistical test: Spearman’s rank-order correlation. P values < 0.05 were considered significant. [file 12916_2020_1529_MOESM4_ESM.pdf]

Supplementary figure 4

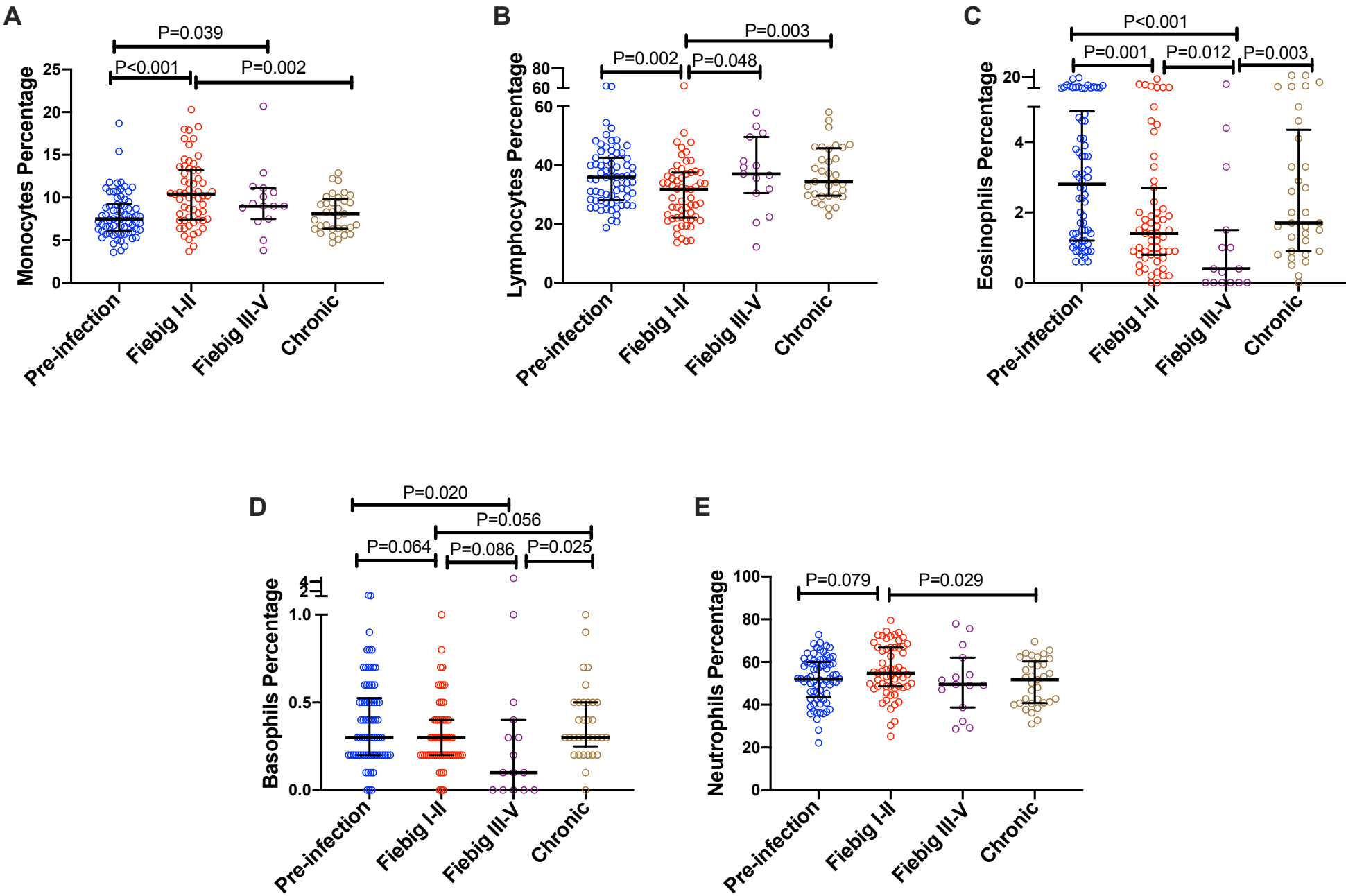

Supplement: Supplementary file 5 — Additional file 5: Supplementary figure 4. Hyperacute HIV infection is associated with dysregulation of proportions of blood lymphoid and myeloid cells. A. Monocytes percentages. B. Total lymphocytes percentages. C. Eosinophils percentages. D. Basophils percentages. E. Neutrophils percentages. For all cellular components, measurements before HIV infection (blue symbols, N = 70), in AHI at Fiebig stage I-II (red symbols, N = 60) and in AHI at Fiebig stage III-V (purple symbols, N = 15) from the FRESH acute infection cohort are shown. Measurements from a different chronic cohort (brown symbols, N = 33) are included for comparison purposes. Each symbol represents an individual participant. Horizontal lines and error bars in scatter plots represent median and interquartile range. Statistical tests used: Wilcoxon rank-sum test (Mann-Whitney U test). P values < 0.05 were considered significant. [file 12916_2020_1529_MOESM5_ESM.pdf]

## Supplementary figure 5

**A**

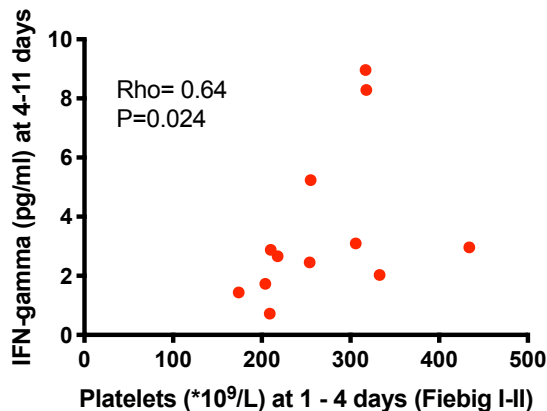

**B**

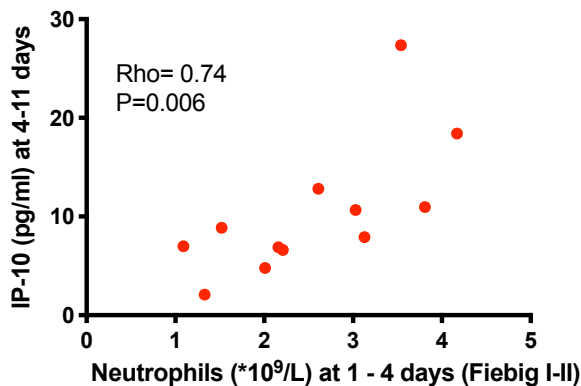

**C**

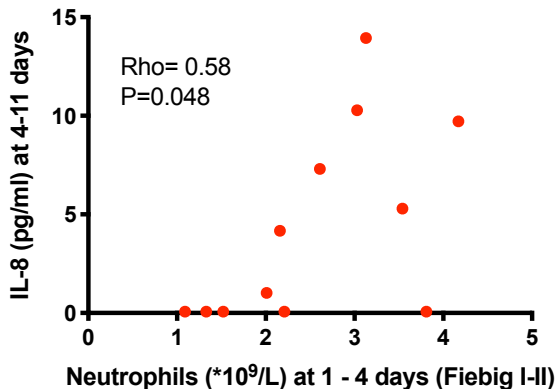

**D**

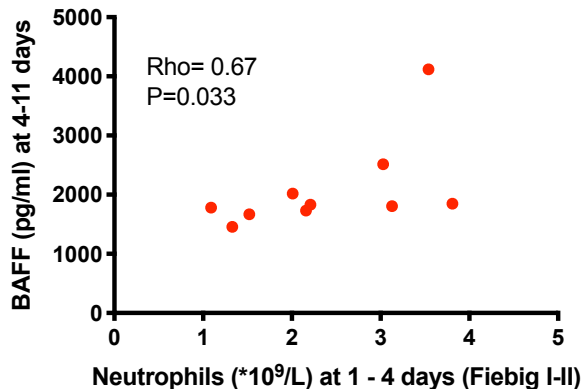

Supplement: Supplementary file 6 — Additional file 6: Supplementary figure 5. Hyperacute plasma cytokines/chemokines are associated with hematological dysregulations. A. Correlation between interferon gamma and platelets. B. Correlation between IP-10/CXCL10 and neutrophils. C. Correlation between IL-8 and neutrophils. D, Correlation between BAFF and neutrophils. The measurements of cytokines and blood cells counts were in the hyperacute phase of HIV infection. Every symbol represents a participant (N = 12 except BAFF with N = 10). Statistical test: Spearman’s rank-order correlation. P values < 0.05 were considered significant. [file 12916_2020_1529_MOESM6_ESM.pdf]
